# Acute macular neuroretinopathy occurrence in a Behçet disease patient: a case report

**DOI:** 10.1186/s12348-025-00457-x

**Published:** 2025-01-13

**Authors:** Ayman Mabchour, Moncef Ould Hamou, Simon Correa, François Willermain, Nacima Kisma

**Affiliations:** 1 Ophthalmology Department, Erasme University Hospital, Centre Hospitalier Universitaire de Bruxelles, Université Libre de Bruxelles, Brussels, Belgium; 2 Ophthalmology Department, Saint Pierre University Hospital, Université Libre de Bruxelles, Brussels, Belgium; 3 Ophthalmology Department, CHIREC Braine-l’Alleud-Waterloo Hospital, Braine l’Alleud, Belgium

**Keywords:** AMN, Acute Macular Neuroretinopathy, Behçet Disease, Posterior Uveitis.

## Abstract

**Purpose:**

To report the occurrence of AMN (Acute Macular Neuroretinopathy) in a Behçet Disease (BD) patient during an active systemic inflammatory relapse and to describe the SD-OCT features of this entity.

**Patients and methods:**

Retrospective observational case report of a patient who presented with an AMN during a BD associated ocular inflammation (Saint Pierre Hospital, Brussels, Belgium). Clinical record and imaging, including infrared reflectance image (IR) and spectral domain optical coherence tomography (SD-OCT), were analyzed.

**Results:**

A 25-year-old man presented with an acute right eye scotoma due to AMN in the setting of active systemic BD. Oral corticosteroid therapy and corticosteroid sparing agents (azathioprine) were prescribed. Close observation revealed progressive lesion regression.

**Conclusion:**

In conclusion, the association between AMN and BD may occur in the context of active systemic disease, though further studies are required to better establish this link. Vigilance appears warranted during inflammatory episodes in BD, as they might contribute to such manifestations. Clinicians could consider BD as a potential differential diagnosis in patients presenting with features suggestive of AMN, and neurological involvement may merit cerebral imaging to exclude other causes. Additionally, the management of posterior uveitis in BD, if present, may benefit from timely and targeted treatment. Further investigations are necessary to refine management strategies for AMN in patients with BD.

## Introduction

Behçet Disease (BD) is a multisystemic relapsing vasculitis typically characterized by the triad of oral aphthosis, genital ulcers and uveitis. The etiology remains unknown.

Historically, the “silk road” region, spanning from the mediterranean to East Asia has been identified as having a higher prevalence of BD [1]. Ocular manifestations are more common and severe in male patients, particularly in early onset disease [2]. Anterior non granulomatous uveitis with hypopyon is a classic presentation, but posterior involvement is the most disabling and sightthreatening form. The latter can encompass papillitis, retinitis, occlusive arteriovenous vasculitis and cystoid macular oedema.

Diagnosis was made from a spectrum of major and minor clinical findings based on the International Study group for Behçet’s Disease [3]. These criteria were revised in 2014 by the International Team for the Revision of the International Criteria for Behçet’s Disease (ITR-ICBD), assigning two points for ocular inflammation, oral aphthous or genital ulcers, and one point for skin lesions, vascular manifestations and central nervous system involvement. A diagnosis of BD is confirmed when a patient accumulates at least four points [4].

Treatment of Behçet’s related uveitis depends on the severity and location of inflammation. Corticosteroids are typically indicated for sight-threatening posterior lesions, often in conjunction with immunomodulatory therapy.

Acute Macular Neuroretinopathy (AMN) is a retinal disorder of unknown etiology. Two distinct subtypes of AMN can be identified. Type 1 AMN, also known as Paracentral Acute Middle Maculopathy (PAMM), affects the inner retinal layers above the outer plexiform layer (OPL). Type 2 AMN, referred to as Typical AMN or Acute Macular Outer Retinopathy (AMOR), impacts the deeper retinal layers below the OPL [5, 6].

AMN is best diagnosed using IR and SD-OCT, which reveal a parafoveal hypointense lesion with sharp-edges and hyperreflective, thickened retinal layers, respectively.

Patients complain of an acute onset paracentral scotoma that eventually fades over time. Microvascular compromise from various origins inducing ischemic retinal injury has been proposed as a pathophysiological pathway, but the etiological process is not clear [7, 8]. We will try to clarify what is known and what remains to be explored on this subject.

## Patients and methods

This is a retrospective observational case report. It complies with the Declaration of Helsinki. The review of the patient’s medical record was approved by the Ethics Committee and by the institutional board of the hospital. The patient provided a written consent.

Clinical records and imaging including infrared reflectance image (IR), spectral domain optical coherence tomography (SD-OCT) and fundus fluorescein angiography (FFA) (HRA Heidelberg Engineering, Germany) were reviewed.

The diagnosis of Behçet’s associated AMN was established based on the patient’s clinical ocular presentation, systemic findings and imaging characteristics.

## Results

### Case description

A 25-year-old man of Mediterranean descent presented to the ophthalmology emergency department complaining of a sudden-onset, painless, unilateral paracentral scotoma in the right eye, noticed a few hours prior.

His history was significant for Behçet’s Disease (BD), diagnosed 10 years ago based on a combination of clinical manifestations (recurrent aphthous ulceration, erythema nodosum, orchitis/epididymitis). We also note an HLA-B51 positivity.

Two weeks prior, the patient experienced a systemic inflammatory flare characterized by fever, symmetric polyarthralgia, oral aphthous ulcers, and erythema nodosum, prompting initiation of methylprednisolone (0.4 mg/kg/day) and colchicine (1 mg/day) by his rheumatologist.

**Fig. 1:**
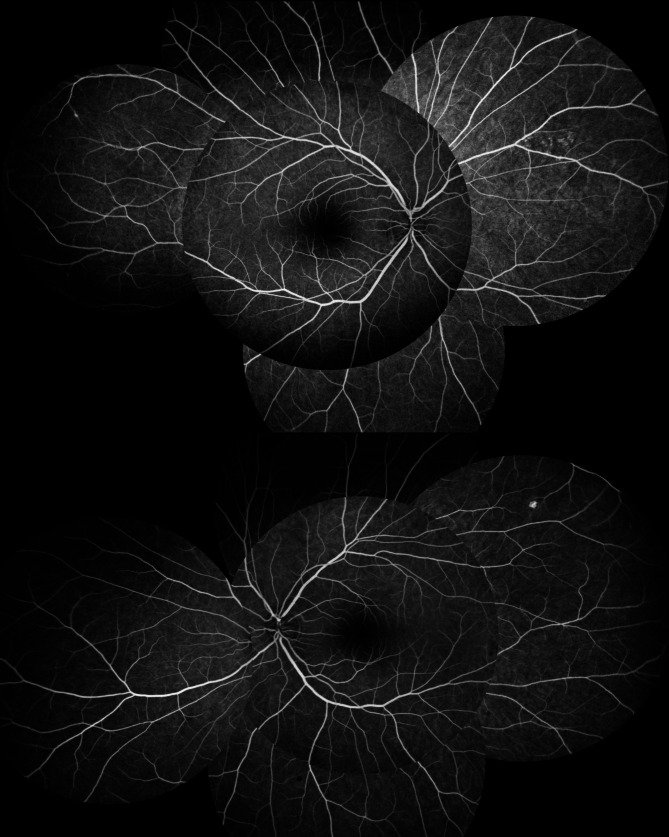
Fundus fluorescein angiography (FFA) at presentation excluding occult vasculitis, papillitis or posterior inflammation. A single peripheral, superotemporal, punched out atrophic lesion was noted on the left eye

Best corrected visual acuity (BCVA) was 20/20 in both eyes. Slit-lamp examination of the anterior segment and anterior vitreous was unremarkable. Fundus examination revealed a single peripheral, superotemporal, punched out atrophic lesion in the left eye but was otherwise unremarkable in the right eye. A fundus fluorescein angiography (FFA Fig. 1) excluded occult vasculitis, papillitis, or any active retinal inflammation.

**Fig. 2:**
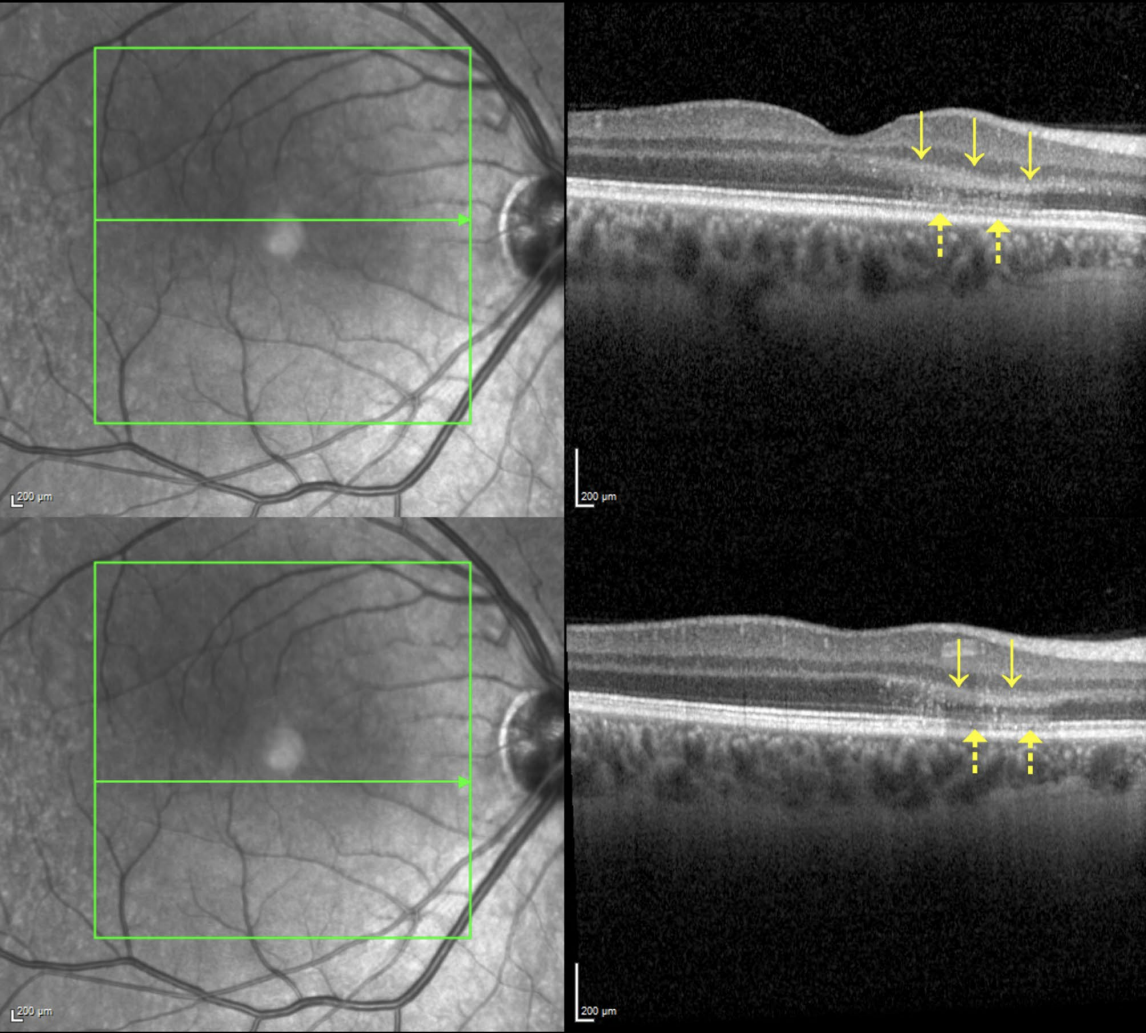
Macular SD-OCT during the acute phase displaying a slight thickening and hyperreflectivity of the outer retinal layers involving the outer plexiform layer and the outer nuclear layer (continuous arrow). The photoreceptor layer and ellipsoid zone were also damaged (dotted arrows)

Macular SD-OCT demonstrated mild thickening and hyperreflectivity of the outer retinal layers, encompassing the outer plexiform layer, the outer nuclear layer, the photoreceptor layer and ellipsoid zone (Fig. 2).

Based on these clinical and imaging findings, The treatment regimen was revised with an increase in methylprednisolone to 0,8 mg/kg/day to better control systemic inflammation and mitigate potential retinal damage and the initiation of azathioprine at a dose of 2 mg/kg/day as a steroid-sparing agent. The rheumatologist also added vitamin supplementation (B12, B6, B9) and anticoagulation therapy (acetylsalicylic acid 80 mg daily).

**Fig. 3:**
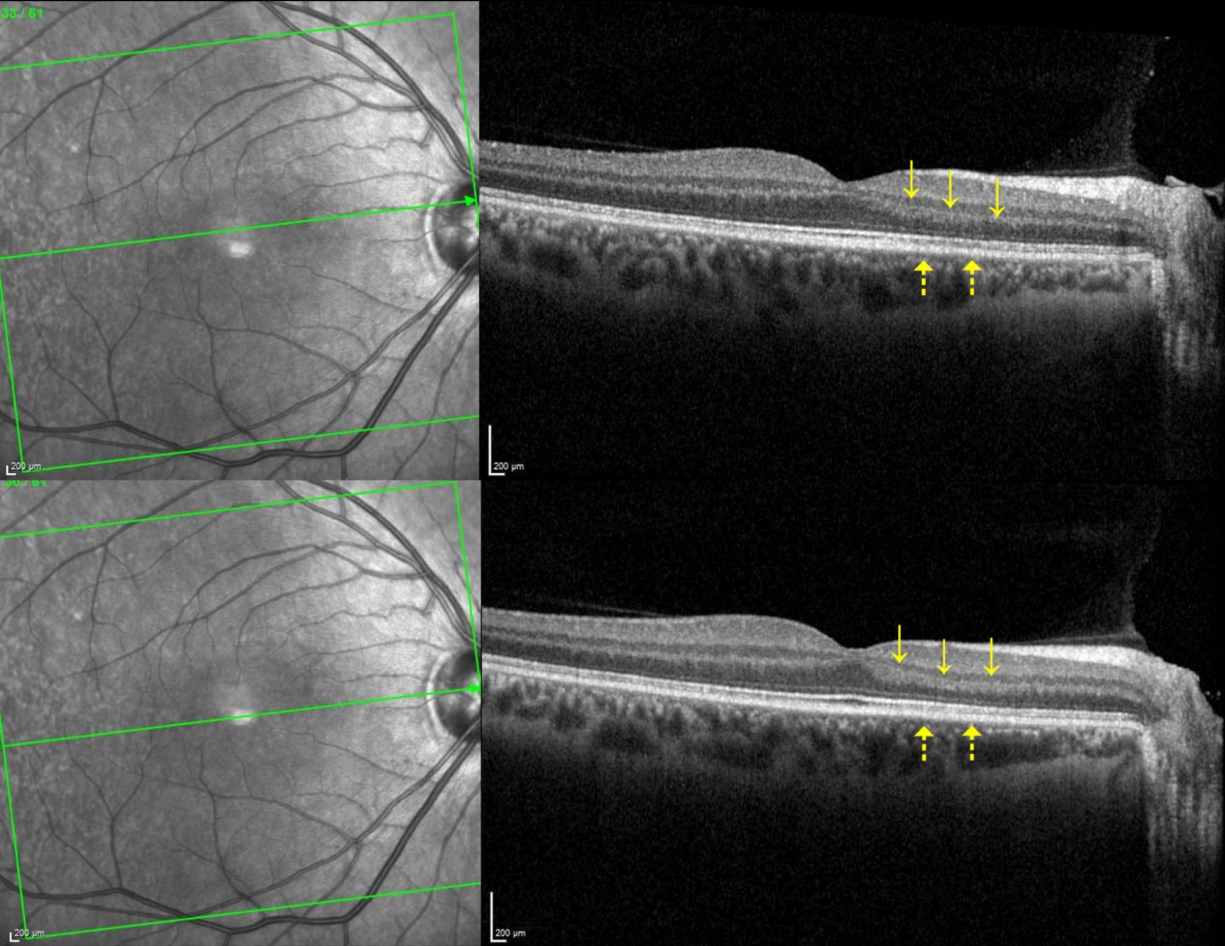
Macular SD-OCT 6 weeks after presentation revealing a resolution of the thickening and hyperreflectivity of the outer nuclear layer (continuous arrow) and a return to a normal photoreceptor layer and ellipsoid zone structure (dotted arrows)

Over six weeks, the scotoma progressively faded, with subjective improvement corroborated by normalization of OCT findings: the thickening of the outer retinal layers involving the outer plexiform layer and the outer nuclear layer normalized (Fig. 3).

Methylprednisolone was gradually tapered. Azathioprine maintenance therapy was tapered and maintained at a dose of 1 mg/kg/day for an indeterminate period following resolution of this episode.

## Discussion

Few cases of AMN or PAMM associated with BD have been reported in the literature. Our case, along with others[10], occurred in a quiet eye, while others [7, 11] were reported in the context of uveitis flare-ups.

Hernanz & al [11] described in 2017 a case series of three young to middle-aged patients who developed AMN during active BD. Two of these patients were undergoing treatment tapering and one of them was treatment free. Concomitant ocular inflammation was noted in all cases : anterior uveitis in two patients, anterior uveitis and vitritis in one, and vitritis and a hot disc in another. All patients presented initially with a scotoma that faded or disappeared over time, consistent with previous reports [7].

Treatment consisted of oral or intravenous corticosteroids, depending on inflammation severity and blinding risk, often in combination with immunosuppressive agents.

Recently Batioglu & al [10] described a 23 year old non-compliant male patient presenting with a several-month history of scotoma. OCT imaging demonstrated thinning of the outer nuclear layers, indicative of a sequelae stage, and a capillary dropout on OCT-A. Multifocal ERG revealed a diminished cone response localized to the AMN region. No evidence of uveitis was identified.

OCT-A was not available at the time of presentation. A post-event OCT-A conducted on our patient revealed no evidence of capillary hypoperfusion.

The persistence of the scotoma and capillary dropout, attributable to delayed presentation, underscores the importance of early diagnosis, the connection between systemic and ocular disease, and the need for specialized, personalized care. Ultimately, patient prognosis is contingent upon these factors.

Very recently, Song & al. [9] reported a case of bilateral PAMM associated with peripheral vascular occlusion in a 58-year-old patient with newly diagnosed BD.

This case emphasizes the importance of considering BD in any patient presenting with an ischemic microvascular event such as AMN and PAMM. A thorough history and physical examination to identify potential signs of BD are essential. Conversely, multimodal imaging should be considered for all BD patients with visual complaints, particularly those with scotomas, to screen for AMN or PAMM and any sign of associated uveitis.

SD-OCT and Infrared Reflectance (IR) imaging are convenient, non-invasive modalities for diagnosing AMN. Humphrey Visual Field (HVF) testing can be valuable assessing functional impairment and patient follow up. While FFA and CFP are ineffective to detect AMN, they can be helpful in identifying associated signs of BD posterior uveitis such as papillitis, vascular sheathing, vasculitis, retinal ischemia and vascular occlusion.

In patients with AMN of unknown etiology, a comprehensive medical history should be obtained, inquiring about oral aphthosis, genital ulcers, arthritis, gastrointestinal discomfort and skin lesions. A multimodal imaging including IR, dense scanning SD-OCT and FFA is recommended to identify occult posterior uveitis associated with BD. A referral to internal medicine to conduct a complete physical examination is advisable.

Additionally, brain imaging (angio-CT or angio-MRI) and cerebrospinal fluid analysis are essential in front of any neurological complaint, including febrile headaches, behavioral disorders or meningeal signs. Papilledema should also prompt further investigations for intracranial hypertension secondary to cerebral thrombophlebitis [12].

Notably, up to 30% of patients with ocular BD develop Neuro-Behçet, which can have severe consequences and significantly impact treatment decisions [13].

Numerous risk factors for AMN have been identified in the literature, including toxic exposure (epinephrine, caffeine), coagulopathies (oral contraceptive, pregnancy state) and hypovolemia [10]. These factors contribute to retinal hypoperfusion and subsequent ischemic macular microinfarction.

BD is a multisystemic vasculitis involving arteries and veins of various sizes, characterized by dysfunction and damage [14]. Acute posterior uveitis in BD typically presents as an arteriovenous occlusive vasculitis with hemorrhages, cotton wool spots andvarying degrees of ischemia. On the microvascular level, this phenomenon can support the hypothesized ischemic pathogenesis process of AMN.

Furthermore BD is a prothrombotic state disease [15] potentially contributing to macular microinfarcts.

Given the progressive nature of BD-associated posterior uveitis without aggressive treatment, we recommend high-dose corticosteroid therapy (oral or intravenous pulse therapy according to severity) combined with a first-line immunomodulatory therapy (IMT), such as azathioprine. Biologic agents should be considered promptly if inadequate response [12].

Our patient experienced an active systemic BD inflammatory flare, necessitating immediate immunosuppressive therapy. Notably, the absence of anterior, intermediate or posterior inflammation suggests a potential thrombotic etiology for the AMN.

While the patient was already receiving anticoagulants by the rheumatologist, the potential benefit of anticoagulation specifically for presumed thrombotic AMN remains an open question.

In conclusion, the association between AMN and BD may occur in the context of active systemic disease, though further studies are required to better establish this link. Vigilance appears warranted during inflammatory episodes in BD, as they might contribute to such manifestations. Clinicians could consider BD as a potential differential diagnosis in patients presenting with features suggestive of AMN, and neurological involvement may merit cerebral imaging to exclude other causes. Additionally, the management of posterior uveitis in BD, if present, may benefit from timely and targeted treatment. Further investigations are necessary to refine management strategies for AMN in patients with BD.

This case report has inherent limitations that must be considered. To begin with, as a descriptive study of a single clinical case, the findings may not be generalizable. In addition, OCT-A was not available at presentation, which limited our ability to evaluate vascular changes in greater detail. Moreover, the absence of multifocal electroretinography (ERGmf) and Humphrey visual field (HVF) testing hindered a more comprehensive assessment of functional impairment and monitoring of the patient’s follow-up.

## Data Availability

No datasets were generated or analysed during the current study.

## Abbreviations

AMN: Acute Macular Neuroretinopathy
BD: Behçet disease
HVF: Humphrey visual fields
FAF: Fundus autofluorescence
FA: Fluorescein angiography
IMT: Immunomodulatory therapy
IR: Infrared reflectance image
SDOCT: Spectral domain optical coherence tomography

## References

[1] Yazici H, Fresko I, Yurdakul S (2007) Behçet’s syndrome: disease manifestations, management, and advances in treatment. Nat Clin Pract Rheumatol 3:148. 10.1038/ncprheum0436 17334337 10.1038/ncprheum0436

[2] Yazici H et al (1984) Influence of age of onset and patient’s sex on the prevalence and severity of manifestations of Behçet’s syndrome. Ann Rheum Dis 43:783–789. 10.1136/ard.43.6.783 6524980 10.1136/ard.43.6.783 PMC1001536

[3] International Study Group for Behçet’s Disease (1990) Criteria for diagnosis of Behçet’s disease. Lancet 335:1078 1970380

[4] International Team for the Revision of the International Criteria for Behçet’s Disease (ITR-ICBD) (2014) The International Criteria for Behçet’s Disease (ICBD): a collaborative study of 27 countries on the sensitivity and specificity of the new criteria. J Eur Acad Dermatol Venereol 28(3):338–347. 10.1111/jdv.12107 23441863 10.1111/jdv.12107

[5] Sarraf D, Rahimy E, Fawzi AA et al (2013) Paracentral acute middle maculopathy: a new variant of acute macular neuroretinopathy associated with retinal capillary ischemia. JAMA Ophthalmol 131(10):1275–1287. 10.1001/jamaophthalmol.2013.4056 23929382 10.1001/jamaophthalmol.2013.4056

[6] Dansingani KK, Freund KB (2015) Paracentral acute middle maculopathy and acute macular neuroretinopathy: related and distinct entities. Am J Ophthalmol 160(1):1–3e2. 10.1016/j.ajo.2015.05.001 26054463 10.1016/j.ajo.2015.05.001

[7] Bhavsar KV, Lin S, Rahimy E, Joseph A, Freund KB, Sarraf D, Cunningham ET Jr (2016) Acute macular neuroretinopathy: a comprehensive review of the literature. Surv Ophthalmol 61(5):538–565. 10.1016/j.survophthal.2016.03.003 26973287 10.1016/j.survophthal.2016.03.003

[8] Acute macular neuroretinopathy, Casalino G, Arrigo A, Romano F, Munk MR, Bandello F, Parodi MB (2019) : pathogenetic insights from optical coherence tomography angiography. Br J Ophthalmol 103:410–414. 10.1136/bjophthalmol-2018-312197 29844084 10.1136/bjophthalmol-2018-312197

[9] Song D, Choi DJ, Bhatt N (2023) Paracentral Acute Middle Maculopathy in Behcet Disease. Retin Cases Brief Rep 1(4):340–342. 10.1097/ICB.0000000000001198 10.1097/ICB.0000000000001198 34618713

[10] Batıoğlu F, Yanık Ö, Demirel S, Özmert E (2021) Multimodal Imaging Characteristics and functional test findings in a case of Acute Macular Neuroretinopathy accompanied by Behçet Disease. Ocul Immunol Inflamm 17(7–8):1424–1430. 10.1080/09273948.2020.1751857 10.1080/09273948.2020.1751857 32510267

[11] Hernanz I, Horton S, Burke TR, Guly CM, Carreño E Acute macular neuroretinopathy phenotype in behçet’s disease. Opthalmic Surg Lasers Imaging Retin. 208;49(8),634–638. 10.3928/23258160-20180803-13 10.3928/23258160-20180803-13 30114310

[12] Kone-Paut I, Barete S, Bodaghi B et al (2021) French recommendations for the management of Behçet’s disease. Orphanet J Rare Dis 24(Suppl 1):352. 10.1186/s13023-020-01620-4 10.1186/s13023-020-01620-4 PMC7903591 33622338

[13] Allegri P, Rissotto R, Herbort CP, Murialdo U (2011) CNS diseases and uveitis. J Ophthalmic Vis Res 6(4):284–308 22454751 PMC3306114

[14] Deuter CM, Kötter I, Wallace GR, Murray PI, Stübiger N, Zierhut M (2008) Behçet’s disease: ocular effects and treatment. Prog Retin Eye Res 27(1):111–136. 10.1016/j.preteyeres.2007.09.002 18035584 10.1016/j.preteyeres.2007.09.002

[15] Seyahi E, Yurdakul S (2011) Behçet’s syndrome and thrombosis. Mediterr J Hematol Infect Dis 3(1):e2011026. 10.4084/MJHID.2011.026 21869912 10.4084/MJHID.2011.026 PMC3152448

